# Burden of hyperphagia and obesity in Bardet–Biedl syndrome: a multicountry survey

**DOI:** 10.1186/s13023-023-02723-4

**Published:** 2023-07-07

**Authors:** Elizabeth Forsythe, Usha G. Mallya, Min Yang, Caroline Huber, Mary Lynn Cala, Alexandra Greatsinger, Ella Hagopian, Jeremy Pomeroy, Andrea M. Haqq

**Affiliations:** 1 Clinical Genetics Department, Guy’s Hospital, and National Bardet–Biedl Syndrome Clinics, Great Ormond Street and St Thomas’ Hospitals, London, UK; 2 Rhythm Pharmaceuticals, Inc., Boston, MA USA; 3 Analysis Group, Boston, MA USA; 4 Marshfield Clinic Research Institute, Marshfield, WI USA; 5 Department of Pediatrics, University of Alberta, 8440 - 112 St NW, Edmonton, AB T6G 2B7 Canada

**Keywords:** Disease burden, Health-related quality of life, Insatiable hunger, School performance, BBS

## Abstract

**Background:**

Signs and symptoms of Bardet–Biedl syndrome (BBS) occur during early childhood, progress over time, and place substantial, multifaceted burden on patients and their caregivers. Hyperphagia may be a contributing factor to early-onset obesity in BBS; however, there are limited insights into its impacts on patients and caregivers. We quantified disease burden as it relates to the physical and emotional impacts of hyperphagia in BBS.

**Methods:**

The CAREgiver Burden in BBS (CARE-BBS) study was a multicountry, cross-sectional survey of adult caregivers of patients with BBS who have had hyperphagia and obesity. The survey consisted of questionnaires including Symptoms of Hyperphagia, Impacts of Hyperphagia, Impact of Weight on Quality of Life (IWQOL)-Kids Parent Proxy, and Patient-Reported Outcome Measurement Information System (PROMIS) v1.0-Global Health 7. In addition, clinical characteristics, medical history, and weight management questions were included. Outcomes were scored and summarized descriptively in aggregate and by country, age, and obesity severity according to weight class.

**Results:**

There were 242 caregivers of patients with BBS who completed the survey. Caregivers observed hyperphagic behaviors throughout the day, with negotiating for food (90%) and waking up and asking or looking for food during the night (88%) being the most frequent. Hyperphagia had at least a moderate negative impact on most patients’ mood/emotions (56%), sleep (54%), school (57%), leisure (62%), and familial relationships (51%). Hyperphagia affected concentration at school (78%), and symptoms of BBS contributed to patients missing ≥ 1 day of school a week (82%). Responses from the IWQOL-Kids Parent Proxy suggested obesity most greatly negatively affected physical comfort (mean [standard deviation (SD)], 41.7 [17.2]), body esteem (41.0 [17.8]), and social life (41.7 [18.0]). On the PROMIS questionnaire, mean (SD) global health score for pediatric patients with BBS and overweight or obesity (36.8 [10.6]) was lower than the general population (mean, 50).

**Conclusions:**

Evidence from this study suggests that hyperphagia and obesity may have broad negative impacts on the lives of patients with BBS, including physical health, emotional well-being, school performance, and personal relationships. Therapies that target hyperphagia may alleviate the extensive clinical and nonclinical impacts experienced by patients with BBS and their caregivers.

**Supplementary Information:**

The online version contains supplementary material available at 10.1186/s13023-023-02723-4.

## Background

Bardet–Biedl syndrome (BBS) is a rare autosomal recessive disease with an estimated prevalence ranging from 1:100,000 to 1:160,000 in Europe and North America [1]. BBS is a genetically heterogeneous syndrome in which patients may develop hyperphagia (insatiable, pathologic hunger), severe obesity, intellectual disability, retinal dystrophy, and renal disease [2–4]. Onset of signs and symptoms in BBS occurs during early childhood, and symptoms progress over time, placing substantial, multifaceted burden on patients with the disease [3, 5].

Obesity, a primary characteristic of the disease, is estimated to occur in 72–92% of patients [2]. Increased food-seeking behavior and rapid weight gain appear during the first years of life and can lead to severe obesity [3, 4, 6]. In a study of patients with BBS, most caregivers reported observing hyperphagic behaviors, such as increased interest in food, before the age of 5 years [4]. Hyperphagia and appetite dysregulation may be a contributing factor to obesity in BBS [7]. Rare variants in BBS-associated genes contribute to impairment of primary cilia, resulting in multisystem dysfunction [8–10] and impairment of the leptin-melanocortin-4 receptor (MC4R) pathway, which may disrupt energy regulation and lead to the increased hunger observed in BBS [7, 11]. Under normal physiologic conditions, leptin binds the leptin receptor on proopiomelanocortin (POMC) neurons in the hypothalamus. The protein encoded by *PCSK1* cleaves POMC to form α–melanocyte-stimulating hormone, which activates MC4R, leading to decreased hunger and increased energy expenditure [12, 13].

Patients with BBS and their caregivers have reported substantial burden, including negative emotional impacts and social life dissatisfaction for patients, which may be a result of the early onset and progressive nature of BBS signs and symptoms [14–16]. Hyperphagia and obesity are considered the most distressing symptoms of BBS [6]. Parents of children with obesity are often stigmatized as the primary individual responsible for the child’s weight, and they have reported healthcare providers as being a major source in reinforcing weight bias/stigma, leading to social blame [15, 17]. In exit interviews following participation in a Phase 2/3 clinical trial, caregivers and patients with BBS reported negative emotional impacts associated with hyperphagia including sadness, frustration, irritability, anxiety, and feelings of guilt [16].

Traditional treatment strategies for obesity due to BBS primarily focus on symptoms and include dietary and exercise management, antiobesity medication, and/or bariatric surgery, which do not target the underlying cause of obesity in this population and have limited effectiveness [2]. Because of the lifelong and progressive nature of BBS, lack of effective treatment options for symptoms is distressing to caregivers and reduces their health-related quality of life [3, 6, 15, 16]. To quantify the unmet need and impact of hyperphagia and obesity in patients with BBS, we devised the CAREgiver Burden in BBS (CARE-BBS) study, a multicountry survey.

## Methods

### Survey fielding and eligibility

The CARE-BBS study was a cross-sectional survey of adult caregivers of patients with a diagnosis of BBS who have had hyperphagia and obesity, which was conducted in the United States, the United Kingdom, Canada, and Germany. Caregivers were recruited through a market research panel. Caregivers of patients with BBS completed a screener to confirm meeting the inclusion criteria, and eligible participants were then invited to complete the survey. Eligible participants must have been ≥ 18 years of age and a caregiver for ≥ 6 months of a patient with BBS of any age who self-reported hyperphagia and either currently had obesity or ever had weight in the 95th percentile or above for their age and sex, as reported by the caregiver. Modified body mass index (BMI) Z score was calculated using Centers for Disease Control and Prevention growth charts according to their methodology [20]. In the pediatric population < 18 years of age, weight classes were defined using percentile-based standards, with overweight being 85th to < 95th percentile, obesity class I being ≥ 95th percentile to < 120% of the 95th percentile, obesity class II being ≥ 120% to < 140% of the 95th percentile, and obesity class III being ≥ 140% of the 95th percentile [21]. For the adult patient population ≥ 18 years of age with overweight or obesity, weight classes were defined using BMI standards, with overweight being BMI 25 to < 30 kg/m^2^, obesity class I being BMI 30 to < 35 kg/m^2^, obesity class II being BMI 35 to < 40 kg/m^2^, and obesity class III being BMI ≥ 40 kg/m^2^ [22]. Professional caregivers who were paid for their time to care for patients with BBS were excluded from the survey. Patients with BBS could not have been enrolled in a clinical trial within 6 months before the survey. Those eligible were able to read and understand the local language of their country and resided in the United States, the United Kingdom, Canada, or Germany at the time of the survey. This study was conducted in accordance with the Helsinki Declaration of 1964 and its later amendments and was granted an exemption from a full review by the United States Pearl Independent Review Board.

### Survey overview and measures

The survey comprised questions on patient clinical characteristics, medical history, and medication management; observer-reported outcome measures and proxy-reported measures; and outcome measures that were used to capture signs, symptom burden, and impact of hyperphagia and obesity on patients and caregivers.

### Symptoms of Hyperphagia—caregiver version observer-reported outcome measure

The newly developed Symptoms of Hyperphagia questionnaire consists of 5 items to measure the frequency of hunger-related behaviors observed by the caregiver in the patient in their care. The questionnaire assesses how often over the past 24 h the person in their care tried to negotiate or argue for more food, ate extremely quickly, sneaked or took food without permission, woke up asking or looking for food during the night, and asked for more food after just finishing a meal or snack. Item responses were “never,” “1 or 2 times,” and “3 or more times” over the past 24 h. The questionnaire is scored as an average across 5 items, with a score range from 0 to 10 and higher scores indicating more severe symptoms of hyperphagia.

### Impacts of Hyperphagia—observer- and self-reported outcome measures

The newly developed Impacts of Hyperphagia questionnaire consists of 2 components, observer- and self-reported, with 10 total items measuring the extent of how hunger behavior affects multiple aspects of life in patients and caregivers, respectively. The Impacts of Hyperphagia caregiver observer-reported version asked caregivers 5 questions on the extent to which hunger negatively affected the person in their care’s sleep, mood or emotions, school, leisure or recreational activities, and relationships with family or friends over the past 7 days. The Impacts of Hyperphagia self-reported version asked caregivers 5 questions on the extent to which the person in their care’s hunger negatively affected their own sleep, mood or emotions, work, leisure or recreational activities, and relationships with family or friends over the past 7 days. The item responses were “not at all,” “a little,” “moderately,” or “a great deal.” The questionnaire components are scored separately for patient impact and caregiver impact, with a score range from 0 to 15, where higher scores indicate greater impacts of hyperphagia.

### Impact of weight on quality of life-kids parent proxy [18]

The Impact of Weight on Quality of Life (IWQOL)-Kids Parent Proxy is a 27-item parent proxy questionnaire assessing health-related quality of life in patients across 4 domains including physical comfort, body esteem, social life, and family relations. Items are reported on a 5-point Likert scale ranging from 1 (always true) to 5 (never true). The total score ranges from 0 to 100, with higher scores representing better health-related quality of life.

### Patient-Reported Outcome Measurement Information System (PROMIS) questionnaire—parent proxy [19]

The PROMIS Parent Proxy Scale v1.0-Global Health 7 is a questionnaire used to examine patient physical, mental, and social health. Scoring, with a possible range from 0 to 100, is based on calculated T scores compared against a mean (standard deviation [SD]) of 50 (10) representing the general population, with higher scores indicating better health.

### Additional survey questions

Additional survey questions on the impacts of hyperphagia and obesity due to BBS were related to weight management medications and strategies, daily meals and caloric intake, and school absenteeism and presenteeism (Supplementary Methods).

### Analysis group classifications and statistical analysis

Descriptive statistics were calculated for each country and in aggregate; instruments were scored and summarized descriptively.

#### Age classification and outcome analysis

Outcomes were also assessed on the basis of age (pediatric, aged < 18; adult, aged ≥ 18); however, 1 caregiver did not specify the age of the patient with BBS in their care resulting in a total sample size of 241 for age analyses.

#### Weight-related classification and outcome analysis

Additional outcomes were assessed according to weight class, which was analyzed in pediatric patients who were identified as having overweight or obesity (n = 224). Because of the small sample size of adult patients (n = 8), weight-related outcomes were not analyzed for this population alone.

## Results

### Respondent demographics and patient characteristics

There were 242 caregivers who met the study criteria and completed the survey across the United States (n = 60), the United Kingdom (n = 59), Canada (n = 62), and Germany (n = 61; Table 1). Caregiver and patient characteristics were generally similar across countries. The mean (SD; range) age of respondents was 41.9 (6.7; 18–77) years, and 54% (n/N = 131/242) were male. Respondents were predominantly parents of the patient in their care (93%; n/N = 226/242), and most were not the sole caretaker of the patient with BBS (82%; n/N = 199/242).

**Table 1:** Caregiver Demographics and Characteristics

	Caregivers (N = 242)
Demographics	
Age, mean ± SD (median; range), y	41.9 ± 6.7 (42; 18–77)
Sex, n (%)	
Male	131 (54.1)
Female	111 (45.9)
Married or in a domestic partnership, n (%)	209 (86.4)
Highest education attainment, n (%)	
High school diploma/equivalent or lower	16 (6.6)
Some college/university or associate’s degree	56 (23.1)
College or university graduate/bachelor’s degree	107 (44.2)
Advanced degree	63 (26)
Household income (in local currency), n (%)	
< 75,000	33 (13.6)
≥ 75,000	208 (86.0)
Prefer not to say	1 (0.4)
Relationship to patient with BBS, n (%)	
Mother	101 (41.7)
Father	125 (51.7)
Other	16 (6.6)
Others responsible for care of patient with BBS, n (%)	
Parent	176 (72.7)
Grandparent	29 (12.0)
Other	32 (13.2)
No others responsible	43 (17.8)
Clinical characteristics; currently receiving treatment for condition (top 5), n (%)	
Eating disorder	19 (7.9)
Anxiety disorder	13 (5.4)
High blood pressure	12 (5.0)
High cholesterol	12 (5.0)
Sleep disorder	10 (4.1)

*BBS* Bardet–Biedl syndrome, *SD* standard deviation

Characteristics and demographics were similar across countries

Survey respondents reported that among the 242 patients with BBS in their care (64% male; n = 155), the mean (SD; range) age was 12 (3.7; 2–30) years with a mean (SD) modified BMI Z score of 4.1 (4.5; Table 2). Overall, 232 of 242 patients (96%) had overweight or obesity (aged < 18 years, n = 224; aged ≥ 18 years, n = 8) at the time of survey response; 55% of pediatric patients (n/N = 123/224) and 88% of adult patients (n/N = 7/8) had class II or III obesity. Symptoms of uncontrollable or insatiable hunger were first noticed at a mean (SD) age of 8.2 (3.6) years and contributed to BBS diagnosis in 230 of 242 patients (95%). Depression (21%; n/N = 50/242), high cholesterol (16%; n/N = 39/242), and sleep disorders (16%; n/N = 38/242) were the most frequently reported past and/or current comorbidities. A large proportion of parents had previously been told by a health care provider or teacher that their child with BBS had a developmental delay (44%; n/N = 106/242). Cognitive impairment was generally reported in a larger proportion of patients with BBS who had obesity class III (behavioral or conduct problem: 41% [n/N = 33/80]; developmental delay: 49% [n/N = 39/80]; learning disability: 45% [n/N = 36/80]; intellectual disability: 40% [n/N = 32/80]; speech or other language disorder: 39% [n/N = 31/80]) compared with the overall population (behavioral or conduct problem: 39% [n/N = 87/224]; developmental delay: 43% [n/N = 97/224]; learning disability: 39% [n/N = 87/224]; intellectual disability: 33% [n/N = 73/224]; speech or other language disorder: 28% [n/N = 62/224]).

**Table 2:** Demographics and Characteristics of Patients With BBS

	Patients (N = 242)
Demographics	
Age at time of survey, mean ± SD (median; range), y	12 ± 3.7 (13; 2–30)
Age categories, n (%), y	
< 6	11 (4.5)
6–11	70 (28.9)
12–17	152 (62.8)
≥ 18	8 (3.3)
Sex, n (%)	
Male	155 (64)
Female	87 (36)
Clinical characteristics	
Modified BMI Z score, mean ± SD (median)	4.1 ± 4.5 (2.8)
Uncontrollable hunger contributed to the diagnosis of BBS, n (%)	230 (95)
Genetic test preformed, n (%)	92 (38)
Family history of genetic obesity, n (%)	52 (21.5)
Age of first BBS symptoms, mean ± SD (median), y	8.4 ± 3.4 (9)
Age categories, n (%), y	
< 6	63 (26)
6–11	120 (49.6)
12–17	48 (19.8)
Age with first noticed symptoms of uncontrollable hunger, mean ± SD (median), y	8.2 ± 3.6 (8.7)
Age categories, n (%), y	
< 6	77 (31.8)
6–11	125 (51.7)
12–17	36 (14.9)
Age of BBS diagnosis, mean ± SD (median), y	8.6 ± 3.5 (9.1)
Age categories, n (%), y	
< 6	64 (26.4)
6–11	139 (57.4)
12–17	36 (14.9)
≥ 18	1 (0.4)
Time between initial BBS symptom onset and BBS diagnosis, mean ± SD (median), y	0.5 ± 0.8 (0.2)
Weight class, n (%)^a^	
Pediatric patients, age < 18 y	
Overweight	27 (12.1)
Obesity class I	74 (33.0)
Obesity class II	43 (19.2)
Obesity class III	80 (35.7)
Adult patients, age ≥ 18 y	
Overweight	0 (0.0)
Obesity class I	1 (12.5)
Obesity class II	5 (62.5)
Obesity class III	2 (25.0)

*BBS* Bardet–Biedl syndrome, *BMI* body mass index, *SD* standard deviation

Characteristics and demographics were similar across countries

^a^In the pediatric population < 18 years of age, weight classes were defined using percentile-based standards, with overweight being 85th to < 95th percentile, obesity class I being ≥ 95th percentile to < 120% of the 95th percentile, obesity class II being ≥ 120% to < 140% of the 95th percentile, and obesity class III being ≥ 140% of the 95th percentile [21]. For the adult population ≥ 18 years of age, weight classes were defined using BMI standards, with overweight being BMI 25 to < 30 kg/m^2^, obesity class I being BMI 30 to < 35 kg/m^2^, obesity class II being BMI 35 to < 40 kg/m^2^, and obesity class III being BMI ≥ 40 kg/m^2^ [22]

### Weight management

**Fig. 1:**
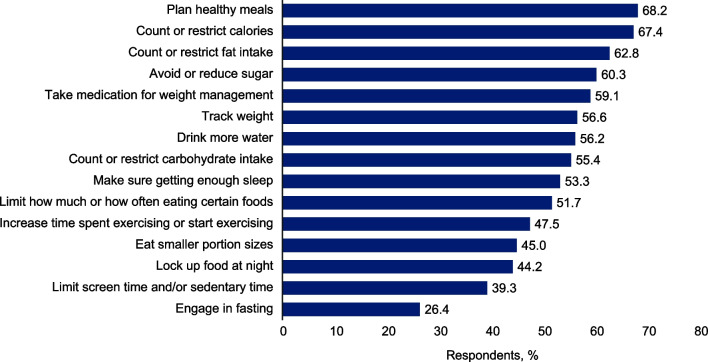
Weight management approaches used by caregivers of patients with BBS at the time of survey

Caregivers reported using an average of 8 weight management approaches for their patient with BBS. The mean (SD) age at which patients with BBS began weight management was 9.2 (3.5) years. The most commonly used weight management strategies at the time of survey fielding were healthy meal planning (68%; n/N = 165/242), restricting calories (67%; n/N = 163/242), restricting fat intake (63%; n/N = 152/242), avoiding or reducing sugar intake (60%; n/N = 146/242), and weight management medication (59%; n/N = 143/242) (Fig. 1). Weight management approaches that were used less frequently included increasing time spent exercising or starting exercising (48%; n/N = 115/242), eating smaller portions (45%; n/N = 109/242), locking up food at night (44%; n/N = 107/242), limiting screen time and/or sedentary time (39%; n/N = 95/242), and engaging in fasting (26%; n/N = 64/242) (Fig. 1). Most caregivers (63%; n/N = 153/242) perceived the patient in their care as moderately or very active.

Patients with BBS were currently using a median of 1 weight management medication, which was consistent across weight classes. Overall, orlistat was the most common weight medication that had been used or was currently being used by 49% of patients with BBS (n/N = 119/242) and 52% of pediatric patients with overweight or obesity (n/N = 116/224). Caregivers expressed an importance for new, effective weight management methods, with a mean (SD) rating of 7.8 (1.4). Overall, the mean (SD) rating for the importance of new, effective weight management methods was similar across all weight classes (overweight, 7.8 [1.3]; obesity class I, 8.2 [1.4]; obesity class II, 7.7 [1.5]; obesity class III, 7.7 [1.3]).

### Symptoms of hyperphagia

**Fig. 2:**
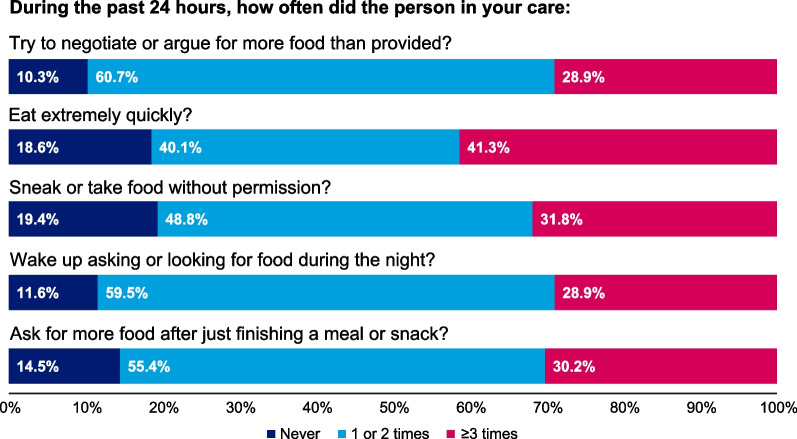
Symptoms of Hyperphagia questionnaire reported by caregivers of patients with BBS

Caregivers of patients with BBS reported observing hyperphagic behaviors throughout the day, with the overall Symptoms of Hyperphagia score increasing by higher weight class (Additional file 1: Table S1). Negotiating for food (90%; n/N = 217/242) and waking up and asking or looking for food during the night (88%; n/N = 214/242) were the most frequently reported signs or symptoms; however, all Symptoms of Hyperphagia behaviors were reported as occurring ≥ 1 time in the past 24 h for > 80% of patients with BBS (Fig. 2). The mean (SD) number of times patients with BBS woke up during the night in the previous 7 days because of insatiable hunger was 6.5 (4.5). Caregivers reported patients with BBS, on average, ate twice as many meals (median, 6 meals) as the other members of the household (median, 3 meals), with 90% (n/N = 217/242) consuming > 2000 cal daily. Of pediatric patients with BBS and overweight or obesity, 91% (n/N = 203/224) consumed > 2000 cal daily.

### Impacts of hyperphagia

**Fig. 3:**
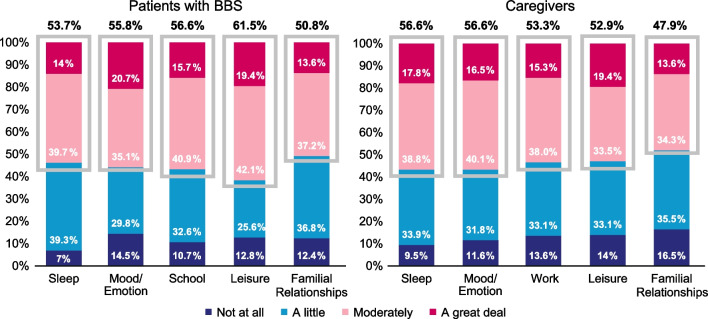
Impacts of Hyperphagia questionnaire results in patients with BBS. Gray boxes and respective percentages indicate cumulative responses for those who were at least moderately affected by hyperphagia. BBS, Bardet–Biedl syndrome

Hyperphagia had at least a moderate negative impact on most patients with BBS across all domains of the Impacts of Hyperphagia questionnaire, including mood/emotion (56%; n/N = 135/242), sleep (54%; n/N = 130/242), school (57%; n/N = 137/242), leisure (62%; n/N = 149/242), and familial relationships (51%; n/N = 123/242) (Fig. 3). More pediatric patients with class II obesity had negative impacts of hyperphagia that either moderately or greatly affected mood/emotions (65%; n/N = 28/43) and school (61%; n/N = 26/43), compared with the overall patient population. More pediatric patients with class III obesity had negative impacts of hyperphagia that either moderately or greatly affected sleep (65%; n/N = 52/80), school (61%; n/N = 49/80), leisure (70%; n/N = 56/80), and familial relationships (56%; n/N = 45/80), compared with the overall patient population. Caregivers of patients with BBS reported impacts of hyperphagia for patients with BBS, with the overall Impacts of Hyperphagia score tending to be higher in more severe weight classes (Additional file 1: Table S1). Caregivers reported that hyperphagia caused difficulty concentrating at school at least sometimes in 78% (n/N = 134/181) of school-aged children with BBS. Further, symptoms associated with BBS caused 82% (n/N = 149/181) of children to miss ≥ 1 day of school a week. School-aged patients with obesity class I and III experienced more difficulty concentrating in school (obesity class I: 83% [n/N = 44/53]; obesity class III: 85% [n/N = 55/65]), and a larger proportion missed ≥ 1 day of school (obesity class I: 83% [n/N = 44/53]; obesity class III: 88% [n/N = 57/65]) compared with all school-aged patients.

### Health-related quality of life

Responses from the IWQOL-Kids Parent Proxy demonstrated obesity had a negative impact on all health-related quality of life domains, with the greatest impact on physical comfort (mean [SD], 41.7 [17.2]), body esteem (41.0 [17.8]), and social life (41.7 [18.0]) in pediatric patients with BBS and overweight or obesity (Table 3). Lower IWQOL-Kids Parent Proxy scores were reported for patients with class III obesity compared with other weight classes (Table 3). On the PROMIS Parent Proxy Scale v1.0-Global Health 7 questionnaire, mean (SD) global health score in pediatric patients with BBS and overweight or obesity (36.8 [10.6]) was lower than the general population (mean, 50). The mean (SD) global health score in patients with BBS was similar across all weight classes (overweight, 38.9 [8.2]; obesity class I, 36.5 [10.4]; obesity class II, 36.6 [10.9]; obesity class III, 36.5 [11.4]).

**Table 3:** IWQOL-Kids Parent Proxy Questionnaire Results in Patients with BBS aged < 18 years with overweight or obesity

	Overall N = 224	Overweight n = 27	Class I n = 74	Class II n = 43	Class III n = 80
Overall	44.2 ± 17.0	52.8 ± 11.1	45.2 ± 17.0	48.9 ± 15.5	37.8 ± 17.3
Physical comfort	41.7 ± 17.2	46.9 ± 11.4	41.2 ± 18.4	46.5 ± 14.8	37.9 ± 18.1
Body esteem	41.0 ± 17.8	47.0 ± 12.4	43.6 ± 18.5	43.8 ± 18.6	35.0 ± 16.9
Social life	41.7 ± 18.0	52.8 ± 11.7	41.8 ± 17.9	46.5 ± 17.6	35.4 ± 17.7
Familial relationships	53.8 ± 25.8	67.6 ± 21.8	54.9 ± 24.3	61.3 ± 22.8	44.0 ± 26.4

*BBS* Bardet–Biedl syndrome, *IWQOL* Impact of Weight on Quality of Life

Values are the mean ± SD. Scores reported are caregiver responses from IWQOL-Kids Parent Proxy and range from 0 to 100, with higher scores representing better health-related quality of life

## Discussion

While hyperphagia may be a substantial contributing factor to early-onset obesity in BBS, its impact on the well-being of both patients and caregivers is poorly understood [4]. In this multicountry survey, caregivers of patients with BBS reported patient burden due to hyperphagia and obesity. In this first real-world study aimed to uncover the physical and emotional impacts of hyperphagia in BBS, hyperphagia played a key role in the diagnosis of BBS (median age, 9.1 years), with 95% of patients presenting with insatiable hunger. Behaviors indicating insatiable hunger were observed amid other symptoms, supporting hyperphagia as an early hallmark of BBS. Thus, early diagnosis of BBS could allow for timely implementation of treatment strategies to target the negative impacts of hyperphagia and obesity in BBS [23].

This study showed that hyperphagia in BBS has a broader impact than merely contributing to the development of obesity. Hyperphagia had a direct and negative impact on school attendance, caregiver-perceived concentration at school, family dynamics, and health-related quality of life of patients. In addition to other symptom-related challenges, hyperphagia contributed to lower school attendance and the inability to focus during school. Exploring relationships between hyperphagia and sleep, school performance, and emotional status would be beneficial to further understand the health-related quality of life burden of BBS.

Severity of obesity was associated with higher levels of health-related quality of life burden in patients. Prior studies have documented the adverse effects of obesity alone on children. A study of adolescents with obesity reported lower IWQOL-Kids scores in more severe weight classes [24]. Similarly, in other PROMIS questionnaire domains assessing health-related quality of life, children with BMI ≥ 99th percentile reported worse anger and fatigue scores than children with lower weight percentiles [25]. In a separate study using PROMIS questionnaire domains assessing health-related quality of life, higher BMI was positively correlated with worse upper and lower extremity physical function, pain interference, and depression [26].

In patients with BBS, the combination of hyperphagia and severe obesity can be particularly damaging for health-related quality of life. Early intervention with effective therapeutics that treat hyperphagia in rare MC4R pathway diseases that lead to obesity may alleviate the symptoms that contribute to lower health-related quality of life and diminished school performance [27].

BBS affects not only the patient but those closest to them including other family members and the primary caregiver [15]. Caregivers of patients with BBS highlighted the need for having new, effective weight management methods. Until recently, obesity due to BBS was treated with conventional approaches including lifestyle modification. Setmelanotide, an MC4R agonist that activates MC4R pathway signaling [28], is an approved treatment option in the United States and European Union for chronic weight management and control of hunger in adult and pediatric patients aged 6 years and older with syndromic obesity due to BBS [29, 30]. Patients with BBS enrolled in a 1-year Phase 3 clinical trial of setmelanotide reported substantial health-related quality of life burden at baseline and clinically meaningful improvement following treatment with setmelanotide [5]. In qualitative interviews, patients with BBS at baseline had hunger that was described as all-consuming and reported improvements in hyperphagia and in physical and/or emotional well-being after setmelanotide treatment [16]. Any improvement in the health-related quality of life of the patient with BBS may translate to improved health-related quality of life for those around them [16].

Strengths of this study included the broad, multicountry scope of respondents and the use of novel questionnaires to probe the effects of hyperphagia in patients with BBS. Because BBS is a rare disease, this large data set provided important critical information on health-related quality of life in patients with BBS of varying backgrounds.

A limitation of this study was the use of proxy measures in the assessment of patients with BBS. Health-related quality of life proxy questionnaires may be influenced from the perspective of the caregiver; however, caregivers are typically knowledgeable about observable signs and behaviors of the patient in their care. Further, given that a large proportion of patients in this survey were pediatric patients and/or reported having intellectual disabilities, we hypothesized the responses from these observer and proxy questionnaires were very close to, if not just as accurate as, information that the patients would report themselves. The current analysis was self-reported by caregivers of patients with BBS who, after meeting eligibility, volunteered to participate in the study. This population may therefore be more astute in recognition of hyperphagia and obesity in BBS than the general population of BBS caregivers. Another limitation of this study is that, although caregivers reported the patients in their care were clinically diagnosed with BBS, not all diagnoses were confirmed.

Further research is needed to assess the generalizability of the results in other geographic settings and the effects of weight management strategies on the burden of hyperphagia. In addition to hyperphagia, the association of other obesity- and BBS-related comorbidities, such as liver steatosis, glucose intolerance, dyslipidemia, and albuminuria, with caregiver and patient burden should be assessed in future studies to determine the best course of treatment. Future studies would also benefit from the analysis of potential correlation between genotype/variant type and severity of hyperphagia and obesity.

Additional CARE-BBS survey components were used to assess the impacts of hyperphagia on caregivers including their global health, work productivity, family life, employment, and financial impact, which will be addressed in a separate publication.

## Conclusions

This study contributes to the unmet need of understanding the impact of hyperphagia and obesity in patients with BBS. The burden of hyperphagia and obesity is significant and broadly affects the lives of patients with BBS, impacting physical health, emotional well-being, school performance, and personal relationships. The health-related quality of life in patients with BBS and their caregivers may be improved by understanding the burdens of insatiable hunger and using therapies that target hyperphagia in BBS.

## Supplementary Information

**Additional file 1. Supplementary Methods: **Additional Survey Questions on the Impacts of Hyperphagia and Obesity Due to BBS. **Supplementary Table 1.** Symptoms and Impact of Hyperphagia in Patients With Bardet-Biedl Syndrome Aged <18 Years With Overweight or Obesity.

## Data Availability

The data sets used and/or analyzed during the current study are available from the corresponding author on reasonable request.

## Abbreviations

BBS: Bardet–Biedl syndrome
BMI: Body mass index
IWQOL: Impact of Weight on Quality of Life
MC4R: Melanocortin-4 receptor
POMC: Proopiomelanocortin
PROMIS: Patient-Reported Outcome Measurement Information System
SD: Standard deviation
