# Supplementary material for: Burden of hyperphagia and obesity in Bardet–Biedl syndrome: a multicountry survey
Source: Orphanet J Rare Dis. 2023 Jul 7;18:182. doi: 10.1186/s13023-023-02723-4 (PMC10327341; doi:10.1186/s13023-023-02723-4)
Supplement: Supplementary file 1 — Additional file 1. Supplementary Methods: Additional Survey Questions on the Impacts of Hyperphagia and Obesity Due to BBS. Supplementary Table 1. Symptoms and Impact of Hyperphagia in Patients With Bardet-Biedl Syndrome Aged <18 Years With Overweight or Obesity. [file 13023_2023_2723_MOESM1_ESM.docx]

**SUPPLEMENTARY MATERIALS**

**Supplementary Methods: Additional Survey Questions on the Impacts of Hyperphagia and Obesity Due to BBS**

*Assessment of Weight Management Medications and Strategies*

Caregivers were queried regarding the specific weight medication that had been or was currently being used and the importance to the caregiver for more effective weight management methods. The importance for more effective weight management methods was rated on a numerical rating scale ranging from 0 (low) to 10 (high).

An item in the survey assessed type and number of current and past weight management approaches used in patients with Bardet-Biedl syndrome (BBS). Approaches evaluated in the survey item included the following: eat smaller portion sizes, count or restrict calories, count or restrict carbohydrate intake, count or restrict fat intake, engage in fasting, drink more water, avoid or reduce sugar, take medication for weight management, increase time spent exercising or start exercising, limit how much or how often eating certain foods, limit screen time and/or sedentary time, lock up food at night, plan healthy meals, track weight, and make sure getting enough sleep.

*Assessment of Daily Meals and Caloric Intake and School Absenteeism and Presenteeism*

Caregivers were queried about the number of daily meals eaten by the patient with BBS in their care and by other family members. The typical daily caloric intake of the patient with BBS was reported in 1 of 4 categories: “1000-2000,” “2001-3000,” “3001-4000,” or “I don’t know.”

Caregivers of school-aged patients with BBS also reported, in the 7 days before taking the survey, how often (in number of days) uncontrollable hunger caused trouble focusing in school and how many days of school were missed because of symptoms of BBS.

| **Supplementary Table 1.** Symptoms and Impact of Hyperphagia in Patients With Bardet-Biedl Syndrome Aged <18 Years With Overweight or Obesity | | | | | |
| --- | --- | --- | --- | --- | --- |
|  | **Overall**  **N=224** | **Overweight**  **n=27** | **Class I**  **n=74** | **Class II**  **n=43** | **Class III**  **n=80** |
| Symptoms of Hyperphagia score (scale range, 0-10) | 5.8 ± 1.8 | 4.9 ± 1.8 | 5.9 ± 1.9 | 5.9 ± 1.6 | 6.2 ± 1.6 |
| Impacts of Hyperphagia patient score (scale range, 0-15) | 7.9 ± 2.9 | 6.5 ± 2.9 | 7.8 ± 3.0 | 7.8 ± 2.7 | 8.5 ± 2.8 |
| Impacts of Hyperphagia caregiver score (scale range, 0-15) | 7.7 ± 3.1 | 6.1 ± 3.1 | 7.8 ± 2.9 | 7.1 ± 2.5 | 8.6 ± 3.4 |

Values are the mean ± standard deviation.
